# The inner life of autism: a call to action

**DOI:** 10.3389/fped.2026.1908533

**Published:** 2026-08-26

**Authors:** Steven Merahn

**Affiliations:** Perimeter Healthcare, Alpharetta, GA, United States

**Keywords:** autism, brain network organization, camouflaging, interoception, intervention, personhood, translational science

## Abstract

Autism intervention systems remain organized around observable behavior, reflecting frameworks established before network neuroscience reshaped how the condition is understood. Meanwhile, adult outcomes in employment, independent living, and social participation remain poor, and intervention trials have rarely followed children far enough to know whether they change. Differences in large-scale brain network organization appear to have reproducible implications for how autistic individuals experience participation, regulate arousal, process internal states, and sustain adaptive performance. This suggests that observable behavior is the downstream expression of network-level organization rather than its primary explanatory locus, with sustained compensatory efforts such as masking and camouflaging carrying measurable costs, including elevated risk for anxiety, burnout, and suicidality. Using joint attention as a case study, this article argues that behavioral description and neural mechanism are not interchangeable, and that training behavioral surface forms without supporting the underlying neural integration may produce assessment-satisfying performance while leaving core developmental functions unaddressed. Rather than asserting a new model of care, the article poses a translational challenge to the pediatric community: to examine whether these network differences should inform the next evolution of autism care, incorporating regulatory capacity, interoceptive awareness, and executive burden as clinical considerations alongside—not merely in service of—observable behavior.

## 1 Introduction

One of the central challenges in translational science is determining when advances in basic science warrant revisiting an established clinical model. Autism may be such a case. Intervention systems continue to be organized primarily around observable behavior, while contemporary neuroscience increasingly describes autism in terms of how the brain is organized at a network level (1, 2). The question is not whether behavior matters, but whether behavior alone remains an adequate foundation for intervention theory.

For more than three decades, applied behavior analysis (ABA) and related behavioral approaches have held a dominant position in autism care, shaping reimbursement structures, training pathways, and definitions of treatment success. The underlying assumption is straightforward: because behavior is observable and measurable, it offers a reliable basis for intervention.

That assumption has served a purpose, but its results invite reflection. While long-term outcomes have been improving over time (3, 4), pooled adult outcome data show that roughly half of autistic adults have poor global outcomes (5), with population-based registry data documenting persistent large gaps in employment, independent living, and social participation (6, 7), and available intervention trials have not extended follow-up to these distal adult domains, with pooled adult employment effects nonsignificant (8). Additionally, independent evidence reviews have raised questions about the strength of the evidence base and the relationship between service intensity and meaningful gains (9). This is not an argument against behavioral methods, but a reason to ask whether the constructs organizing care capture what matters most for how these children fare across the life course.

Meanwhile, understanding of the neural mechanisms underlying autism has grown dramatically. Converging evidence suggests autism may be better understood not simply as a behavioral syndrome with neural correlates, but as a neurodevelopmental phenotype characterized by distinctive patterns of large-scale brain network organization (1, 2). These differences appear to influence how individuals perceive, prioritize, regulate, and interpret experience, such that observable behavior may represent, in part, the downstream expression of network-level organization rather than its primary explanatory locus (10).

A word on how to read what follows. The findings described here are, at this stage, largely candidate mechanisms rather than validated intervention targets. The aim is not to claim that we can yet act on them, but to suggest that they are substantial—and clinically resonant—enough to deserve a closer look. Where the language points toward intervention, it points toward questions worth investigating, not conclusions already reached.

The translational question is therefore simple: if autism is increasingly understood at the level of network organization, what might follow for how we care for autistic children and their potential life-course outcomes?

## 2 What the networks might be telling us

Several large-scale brain networks show reproducible organizational differences in autism. Four are offered here as examples, each chosen because it connects to something clinicians already observe.

Default mode network. Many clinicians know the child who seems, at moments, to be “in their own world”—present but inwardly absorbed. The default mode network (DMN) supports self-referential, inwardly directed processing and is normally suppressed during tasks requiring external attention. In autism, that suppression appears reduced, so inner experience may remain more continuously active (11). This suggests an alternative organization of self-experience rather than an absence of social awareness—with implications for how a child experiences participation, meaning, and connection.

Salience network. What looks from the outside like inattention or disengagement may, from the inside, be a different weighting of what matters. The salience network arbitrates which internal and external signals capture attention; in autism, interoceptive and internally generated information often appears to be prioritized differently than in neurotypical peers (11). Some salience network measures have even shown the capacity to distinguish autistic from non-autistic children and to gauge symptom severity, an indication that this research finding may have clinical application (12).

Interoception and the insular cortex. Consider the child who does not recognize the rising discomfort that precedes urinary incontinence or emotional dysregulation, or doesn't acknowledge another person in pain. Interoception—the perception of internal bodily states—is the substrate for recognizing and communicating emotion and physical sensation, and interoceptive differences are among the more robustly characterized features of autism in aggregate analyses (13). They have been linked to alexithymia, dysregulation, and anxiety, though the neural picture is still being worked out and individual studies are mixed (14). The clinical relevance does not depend on any single mechanism: when internal cues escalate quickly or are poorly differentiated, the window for conscious regulation narrows, and interventions that emphasize externally reinforced emotional control may promote suppression rather than genuine regulatory capacity. Notably, interoception is already a testable target—a randomized controlled trial of interoceptive training reduced anxiety in autistic adults (15)—which is exactly the kind of question a translational agenda could pursue.

Frontoparietal control network. Perhaps the most clinically familiar pattern is the child who holds together all day at school and falls apart the moment they get home, or perhaps at lunch or the moment the class bell rings. The frontoparietal network (FPN) supports executive control and top-down regulation, and in autism it often appears to work overtime, compensating for differences in salience, interoception, and self-referential processing (16). Sustained compensation of this kind—monitoring one's own behavior, suppressing natural responses, performing social fluency—has a cost. Accumulating over time, it corresponds to what the literature calls masking or camouflaging, which appears to be driven by executive load and perceived social misfit rather than by intellectual ability (17), and which is increasingly associated with anxiety, burnout, and suicidality, even as the causal pathways are still being delineated (18–20).

Taken together, these patterns suggest that some of the clinically important machinery of autism resides not only in behavior, but in the organization and interaction of large-scale neural systems. If these systems prove to participate in learning, regulation, and adaptation—as increasingly seems plausible—they become candidate targets worth investigating, not merely frameworks for explanation.

## 3 Joint attention as a case study

Joint attention illustrates why behavioral description and neural mechanism are not interchangeable.

In behavioral frameworks, joint attention is operationalized through observable acts—gaze shifts, showing, pointing. Because these predict later language and social development, intervention often works to increase their frequency and consistency.

Neuroscience suggests something more layered. Joint attention appears to emerge from coordinated information processing across systems for self-representation, social representation, and attention—including interactions between DMN and salience networks (10)—and can be present even in the absence of observable expressions (21). The defining feature is less a specific motor act than the capacity to internalize shared experience within a common frame of reference.

That capacity is foundational to early co-regulation, through which children learn to modulate arousal and emotion within relationships (22). A purely behavioral approach—one that elicits the observable response without supporting the underlying relational and cognitive process—may not adequately support the neural integration through which shared experience becomes meaningful (23).

The broader point: an autistic child may genuinely share attention while expressing it differently from what standard tools are built to detect.

## 4 From mechanisms to a question about intervention

The implications reach beyond any single network. Salience allocation, interoception, self-referential cognition, and executive control operate as an integrated system shaping how autistic children learn, regulate, and adapt. If these mechanisms prove to contribute to developmental outcomes, they become potential targets worth investigating rather than explanatory constructs alone.

None of this argues for abandoning behavioral methods. They remain valuable for teaching functional skills, supporting communication, and addressing safety (6), and contemporary autism care at its best is already multidisciplinary, family-centered, and guided by developmental context. The question is not whether behavior matters, but whether behavioral outcomes alone are sufficient indicators of functional and developmental progress.

A network-informed lens suggests a shift in emphasis. Regulatory capacity might be seen as a prerequisite for learning rather than a secondary concern; interoceptive awareness a potential intervention target; executive burden a clinical variable rather than an invisible cost. And many strengths associated with autism—perceptual precision, systematic thinking, sustained focus—appear to arise from the same architecture as its challenges (24), which cautions against treating every feature of autistic cognition as an impairment to be corrected. These would be candidate targets to be assessed and applied where indicated in the individual, not universal protocols. The heterogeneity of autistic experience is precisely why individualized assessment, rather than uniform intervention, is the appropriate translational path—a stratified logic developed more fully elsewhere (9).

The underlying principles of developmental, relationship-based intervention (DRBI) frameworks—among them DIR/Floortime, SCERTS and the Play Project—are broadly consistent with contemporary mechanistic accounts. Rather than targeting behavioral surface forms, they address the regulatory, relational, and representational capacities from which functional development proceeds. The rationale for these targets is now substantially clearer than it was when the behavioral paradigm was institutionalized—and what neuroscience increasingly provides is a principled basis for why they deserve rigorous investigation.

What these ideas mean in practice shifts with development. In early childhood, the emphasis falls on co-regulation within caregiving relationships; in the school years, on the mounting executive load of masking in demanding environments; in adolescence and the transition to adulthood, on the outcomes—participation, autonomy, mental health—that the current system too often fails to secure. A pediatric framework has to hold all of these in view.

## 5 The translational challenge

A neuroscience-informed framework would ask more than whether a child produced or reduced a target behavior. It would ask whether intervention supported underlying neural mechanisms associated with regulation, eased executive burden, enhanced interoceptive access, supported participation, and improved long-term well-being. In the exam room, these translate into questions any clinician can recognize: does this child have a friend? Do they take part in something they value? Do they have some say over their day? Do they feel, and function, better over time? These are candidate outcome domains a research agenda would need to define and validate—offered here as illustrations, not as a finished measure set.

It is worth being candid about the limits of the current evidence. The findings above are largely correlational; they describe group-level patterns, not a single, uniform “autistic brain,” and autistic individuals vary widely from one another and across time. Other mechanistic accounts—predictive coding, excitation/inhibition balance, neural noise—offer complementary and often convergent framings rather than competitors. And important questions about the reliability of network measures across methods, and their meaning for any individual child, remain open. These uncertainties are not a reason to wait; they are the substance of the work this article calls for.

The gap is fundamentally translational. Scientific understanding has moved toward network-level explanation, while reimbursement, training, outcome measures, and service systems remain aligned with earlier behavioral models. Basic science has, for now, outpaced its integration into care.

## 6 Closing the gap

Translational science succeeds only when understanding changes practice. For autism, the question is no longer whether large-scale network differences exist; converging evidence implicates brain network organization in shaping autistic development and experience. The open question is what intervention theory should do with that knowledge.

The pediatric community is well positioned to explore this opportunity. The next step is to determine which intervention targets, developmental supports, and outcome measures follow from a network-informed view—moving beyond metrics of behavioral performance alone toward measures that also capture regulation, interoceptive competence, adaptive functioning, well-being, and developmental trajectory. These align naturally with neuro-affirming care, which treats neurological difference as a basis for adapted support rather than a target for normalization (25).

Medicine has many examples of clinical models that outlived the assumptions on which they were built. Autism intervention may be nearing such an inflection point. Network neuroscience has begun to describe, in increasing detail, how autistic children perceive, regulate, learn, and participate. The challenge now is not to prove these mechanisms exist, but to ask what follows from them. If intervention science is to stay aligned with our understanding of the condition it addresses, the next generation of autism care should be informed not only by how autistic children behave, but by how autistic minds work.

## Data Availability

The original contributions presented in the study are included in the article/Supplementary Material, further inquiries can be directed to the corresponding author.
